# Cataract Progression Associated with Modifications in Calcium Signaling in Human Lens Epithelia as Studied by Mechanical Stimulation

**DOI:** 10.3390/life11050369

**Published:** 2021-04-21

**Authors:** Marko Gosak, Dajana Gojić, Elena Spasovska, Marko Hawlina, Sofija Andjelic

**Affiliations:** 1 Faculty of Medicine, University of Maribor, 2000 Maribor, Slovenia; marko.gosak@um.si; 2 Faculty of Natural Sciences and Mathematics, University of Maribor, 2000 Maribor, Slovenia; 3 Eye Hospital, University Medical Centre, 1000 Ljubljana, Slovenia; g.dajana@gmail.com (D.G.); elena.spasovska90@gmail.com (E.S.); marko.hawlina@kclj.si (M.H.)

**Keywords:** human eye lens, lens epithelial cells, calcium signaling, mechanical stimulation, calcium waves, cataract, intercellular communication, paracrine signaling

## Abstract

Ca^2+^ homeostasis and signaling disturbances are associated with lens pathophysiology and are involved in cataract formation. Here, we explored the spatiotemporal changes in Ca^2+^ signaling in lens epithelial cells (LECs) upon local mechanical stimulation, to better understand the LECs’ intercellular communication and its association with cataractogenesis. We were interested in if the progression of the cataract affects the Ca^2+^ signaling and if modifications of the Ca^2+^ homeostasis in LECs are associated with different cataract types. Experiments were done on the human postoperative anterior lens capsule (LC) preparations consisting of the monolayer of LECs on the basement membrane. Our findings revealed that the Ca^2+^ signal spreads radially from the stimulation point and that the amplitude of Ca^2+^ transients decreases with increasing distance. It is noteworthy that a comparison of signaling characteristics with respect to the degree of cataract progression revealed that, in LCs from more developed cataracts, the Ca^2+^ wave propagates faster and the amplitudes of Ca^2+^ signals are lower, while their durations are longer. No differences were identified when comparing LCs with regard to the cataract type. Moreover, experiments with Apyrase have revealed that the Ca^2+^ signals are not affected by ATP-dependent paracrine communication. Our results indicated that cataract progression is associated with modifications in Ca^2+^ signaling in LECs, suggesting the functional importance of altered Ca^2+^ signaling of LECs in cataractogenesis.

## 1. Introduction

The ocular lens needs to be transparent to transmit and focus light to the retina. Cataracts are opacities of the lens and are the most common cause of vision impairments. Cataracts caused 33.4% of all blindness worldwide in 2010 (10.8 million people) and 18.4% of all moderate to severe vision impairments (35.1 million people) [1].

Structurally, the lens has three main parts: the basement membrane completely surrounds the lens; the single-layered lens epithelium, built from the lens epithelial cells (LECs), is located in the anterior portion of the lens between the basement membrane and the lens fibers; the lens fiber cells form the bulk of the lens. Based on the region of opacification, cataracts are mainly of three types: nuclear (N), located in the center of the lens; cortical (C), located in outer layers of the lens and posterior subcapsular cataracts (PSC). Combined, cortical and nuclear (C&N) cataracts, are also often present. Moreover, cataracts are classified according to their degree of development as well.

The lens epithelium on the basal lamina is the first physical and biological barrier in the lens between the aqueous humor and the lens fiber cells. It is metabolically the most active part of the lens, sustaining the physiological health of the tissue. The cells of the lens epithelium regulate most of the homeostatic functions of the lens [2]. The role of the lens epithelium in the pathogenesis of cataract was studied already in the early 1960s [3]. 

An important question in lens epithelium research is the role of the altered intercellular signaling (including Ca^2+^ signaling) in LECs and the subsequent effect this may have in cataract formation [4,5]. Intercellular communication is essential for the coordination and synchronization of cellular processes. Cell signaling is part of any communication process that governs basic activities of cells and coordinates all cell actions. Calcium, Ca^2+^, is a universal intracellular messenger involved in essential cellular functions and it is a key mediator of signaling within lens cells [6,7]. The role of LECs in controlling the lenticular Ca^2+^ is of interest, since mature lens fibers have no organelles or nuclei, so they do not possess intracellular Ca^2+^ stores such as the endoplasmic reticulum and mitochondria [8,9,10]. Nevertheless, it should be noted that differentiated lens fiber cells, which are developing from LECs, still have nuclei, organelles [8,10], and Ca^2+^ signaling capabilities [7,11,12].

Free cytoplasmic Ca^2+^ concentration, [Ca^2+^]_i_, in LECs is always kept low under physiological conditions [7]. The duration and the magnitude of [Ca^2+^]_i_ elevation is generally very tightly regulated. Gupta et al. [9] (2004) reported that in the LECs from the central zone of epithelium, the total Ca^2+^ levels are always several-fold higher for the lenses with the cataract than in those taken from the clear controls, irrespective of the type of cataract. In our previous study of Ca^2+^ signaling in LECs after acetylcholine stimulation, we also did not detect any significant differences in any aspect of Ca^2+^ signaling between lens capsules (LCs) from different types of cataracts (cortical/nuclear). However, we have identified considerable differences with respect to the stage of the cataract. In particular, LCs associated with more developed cataracts were found to exhibit a slower collective response to stimulation and a less pronounced spatiotemporal clustering of Ca^2+^ dynamics in LECs. Our previous findings indicate that the cataract progression entails the impairment of intercellular signaling [13], suggesting the functional importance of altered Ca^2+^ signaling of LECs in cataractogenesis.

Moreover, we and others have studied the structural and functional features of LECs and their connections. In previous studies, we provided detailed evidence about the structural organization of the anterior LECs, showing the extensions and the entanglements of the LECs cytoplasmic membrane at the border with the basal lamina and showing the gap junctions laterally between the adjacent LECs [14]. We also studied the ex vivo cultured human LECs and have shown the formation of lateral connection between the cells [15]. We studied the contractions of anterior LCs LECs and their association with the Ca^2+^ dynamics of the cells. During the contraction, the LECs remain connected at several locations, presumably gap junctions. The process of contraction is reversible [16].

In this work, the starting postulate was that the cells have the ability to perceive and correctly respond to their microenvironment to maintain normal tissue homeostasis. Between many different cell types, the intercellular communication is mediated through gap junctions and/or through the release of paracrine mediators. Errors in intercellular signaling interactions and cellular information processing are often responsible for diseases [17,18]. Ca^2+^ spread reflects the intercellular communication, and the intercellular Ca^2+^ signaling in LECs may play a role in regulating cytosolic Ca^2+^ in the intact lens [19]. Abnormalities in Ca^2+^ signaling in LECs are implicated in the development of cataracts [7,20]. Intercellular Ca^2+^ propagation evoked by a local mechanical stimulus is one of the assays to study intercellular communication. In experimental settings, intercellular Ca^2+^ propagation can be elicited by applying a mechanical stimulus to a single cell. For example, mechanical stimulation was used in ovine cultured LECs to study the regulation of lens Ca^2+^, and intercellular communications were shown to be governed by gap junctions: when gap junctions were uncoupled, the increase in Ca^2+^ was almost entirely limited to the mechanically stimulated cell [19]. Notably, it has been reported that lens cell sensitivity to mechanical stimulation may be linked to traumatic cataracts, in which physical injury of the lens results in opacity [21].

The purpose of this study is to explore and identify intra- and intercellular Ca^2+^ signaling in human anterior LECs upon local mechanical stimulation, in order to better understand the role of Ca^2+^ in intercellular communications related to cataract formation. More specifically, we were interested if the progression of the cataract (mild or moderate) affects Ca^2+^ signaling and if modifications of the Ca^2+^ homeostasis in LECs are associated with different types of cataract (cortical or nuclear). Moreover, we examined if the Ca^2+^ wave spreading phenomenon depends on paracrine ATP signaling. Spatial changes in intercellular Ca^2+^ concentration following mechanical stimulation were measured in whole monolayers of LECs. In our analyses, we particularly investigated the characteristics of Ca^2+^ signaling with regard to the distance from the point of mechanical stimulation in different subgroups referring to the stage of cataract progression and the cataract type. To determine if in human lens epithelium intercellular Ca^2+^ waves initiated by mechanical stimulation are mediated by gap junctions, paracrine ATP signalization or both and in what extent, we excluded the paracrine ATP component by enzymatic degradation of ATP by the ATP-hydrolyzing enzyme, Apyrase. To the best of our knowledge, the role of paracrine ATP intercellular signaling has not been studied in the context of Ca^2+^ wave propagation in human LC LECs.

## 2. Materials and Methods

### 2.1. Ethics Statement

The research followed the tenets of the Declaration of Helsinki. The study was approved by the National Medical Ethics Committee of the Republic of Slovenia (protocol code 0120-577/2017-7 and date of approval 31 October 2017) and all patients signed informed consent before the operation.

### 2.2. Lens Capsules Preparation

Experiments were done on the anterior LC preparations consisting of the monolayer of LECs attached to the basement membrane, i.e., the capsule matrix. The LCs were obtained routinely during cataract surgery performed at the Eye Hospital, University Medical Centre (UMC), Ljubljana, Slovenia. The central LECs were studied from the approximately 5–5.5 mm circles of the central anterior LCs that were carefully removed by continuous curvilinear capsulorhexis. The material originated from 13 different patients whose age was between 35 and 82, with the average being 69 years. A detailed overview is provided in Table 1. PEX, diabetes, uveitis, trauma or other causes of cataracts, except for age-related causes, were excluded. We systematically analyzed different aspects of intra- and intercellular Ca^2+^ signaling in the human anterior lens epithelium, built from LECs, whereby we compared different signaling characteristics with regard to the degree of cataract progression. The grading was based on modified LOCS III system [22], where 1 is the lowest and 5 is the highest level of cataract development, and to the type of the cataract as cortical (C), nuclear (N), and combined cortical and nuclear (C&N). Combinations of mixed cataracts, not specifically classified in LOCS III system, were subjectively evaluated according to the same grading system of 1–5. As no differences were found between different types of cataracts, the results from either type were pooled and noted as CN. Further, we designated stages 1 and 2 as mild and stages 3–5 as severe cataracts and focused on differences between mild (CN1–2) and severe (CN3–5) cataracts, irrespective of the type of cataract. In the analyses, 34 experiments on different LCs’ regions were included, 10 originating from patients with cortical cataracts, 17 from nuclear cataracts, and 7 from mixed cataracts. With regard to the stage of the cataract, 17 of them were categorized as mild cataracts, 13 as severe cataracts, and in only 3, the type and not the stage were determined, and these were excluded from the cataract stage comparison. After the surgery, each LC was stored in a high glucose medium (DMEM; Sigma, No. 5671, St. Louis, MO, USA) supplemented with 10% FBS and 1% antibiotics (penicillin–streptomycin; Sigma, No. 4333), and transported at a room temperature to the research laboratory in the same building in approximately half an hour. Until utilization, the LCs were kept in a CO_2_ incubator (Innova CO-48; New Brunswick Scientific, Enfield, CT, USA) at 37 °C and 5% CO_2_. The LCs were loaded with the AM ester of Fura-2 (Fura-2 AM; Invitrogen–Molecular Probes, Waltham, MA, USA). For loading Fura-2 AM in DMSO was suspended in 3 mL MEM to a final concentration of 2μM. The loading was done in the incubator at 37 °C for 30 min. After loading, the LCs were washed twice for 7 min with MEM. LCs were then transferred to the plastic glass bottom Petri dishes (Mattek Corp., Ashland, MA, USA; 3.5 cm in diameter) filled with 3 mL of the bath solution with (in mM): NaCl 131.8, KCl 5, MgCl_2_ 2, NaH_2_PO_4_ 0.5, NaHCO_3_ 2, CaCl_2_ 1.8, HEPES 10, and glucose 10, at pH 7.24. LCs were gently stretched by using microdissecting tweezers (WPI by Dumont, Med. Biologie, Friedberg, Germany), and then they were immobilized by a harp-like grid, a parallel array of fine nylon monofilament threads glued at approximately 500 µm intervals to a U-shaped platinum wire frame, similar to the one used for experiments with small vertebrate brain slices [23], so that the mechanical stimulation would not displace them. The grid also flattened the LC, which was necessary for the optical recording. The orientation of the LC was with the basement membrane to the bottom, so the mechanical stimulus was applied to the LECs and not to the basement membrane. The Petri dish with the immobilized LC was then mounted on the inverted microscope Zeiss Axiovert S 100 (Carl Zeiss, Jena, Germany). To test responses to mechanical stimuli, the mechanical stimulation with a tip of a glass micropipette mounted on a MP-285 micromanipulator (Sutter, Novato, CA, USA) was used. A glass micropipette was made by pulling a glass capillary (no. TW150F-3, World Precision Instruments, Sarasota, FL, USA) with a puller (P-97 Sutter Instrument, Novato, CA, USA). Prior to use, the tip of the pipette was heat-polished until it rounded up (Micro-Forge, MF-200, World Precision Instruments, USA). The micropipette was placed over a single cell and mechanical stimulation was applied to the cell by lowering the micropipette onto the surface of the cell until touching it, and then rapidly returning it to its original position. Different regions of the same LC were mechanically stimulated.

To study the paracrine effect of ATP on signal spreading, an additional 5 LCs were studies after the control mechanical stimulation experiment recording, incubated for 30 min with Apyrase (A7646, Sigma, St. Louis, MO, USA) at 3 µL in 2.5 mL of physiological solution, and then again stimulated mechanically with a micropipette, so that the effect of Apyrase was compared on the same LC. Apyrase is an enzyme which hydrolyzes ATP and effectively removes ATP from the extracellular milieu. The material originated from 5 different patients whose age was between 63 and 83 with the average being 74 years (see Table 1).

### 2.3. Calcium Imaging

Image acquisition was done with the 12-bit cooled CCD camera SensiCam (PCO Imaging, Kelheim, Germany). The software used for the acquisition was WinFluor (written by J. Dempster, University of Strathclyde, Glasgow, UK). Objectives used were: 40X/0.75 Plan-NeoFluar and 63X/1.25 oil Plan-NeoFluar (Zeiss, Jena, Germany). The light source used was XBO 75 W (Zeiss, Germany) Xe arc lamp. The light intensity was attenuated when necessary with grey filters with optical densities of 0.5, 1, and 2 (Chroma, Foothill Ranch, CA, USA). The excitation filters used, mounted on a Lambda LS-10 filterwheel (Sutter Instruments Co., Novato, CA, USA), were 360 and 380 nm (Chroma, Foothill Ranch, CA, USA). Excitation with the 360-nm filter (close to the Fura-2 isosbestic point) allowed for the observation of the cells’ morphology and of the changes in the concentration of the dye, irrespective of changes in [Ca^2+^]_i_, while the 360/380 nm ratio allowed for the visualization of the Ca^2+^ concentration changes in the cytoplasm. Image acquisition, timing, and filter wheel operation were all controlled by WinFluor software via a PCI6229 interface card (National Instruments, Austin, TX, USA). The criteria for selecting the regions for imaging were the presence of adherent cells and good cell morphology, both assessed by observation of transilluminated and 360-nm fluorescent images. Several repetitions of the mechanical stimulation on the different regions of on the same LC were done. Image frames were acquired every 500 ms, resulting in frame cycles being 1 s long (alternating 360 nm and 380 nm recording). The manual selection of individual cells (regions of interest, i.e., ROI) and the subsequent exportation of Ca^2+^ traces were performed with ImageJ software (National Institutes of Health, Bethesda, MD, USA).

### 2.4. Characterization of Calcium Dynamics

**Figure 1:**
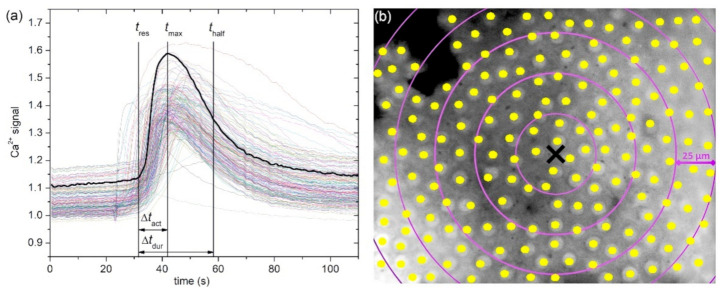
Characterizing the spatiotemporal Ca^2+^ activity in LECs. (**a**) Traces of intracellular Ca^2+^ dynamics (ratio 360/380) in all 180 LECs in a typical LC epithelium. The vertical lines and arrows indicate the relevant time of interest for a given LECs (thick black line): response time, *t*_res_; time at which the maximal amplitude was reached, *t*_max_; the half-decay time, *t*_half_; the activation time, Δ*t*_act_; and the signal duration time, Δ*t*_dur_. (**b**) Image of the LC epithelium recorded with 360 nm. Yellow dots denote selected LECs, i.e., midpoints of ROIs. The black cross shows the location of the mechanical stimulation. Purple circles indicate the concentric regions divided by annuluses, each 25 µm more distant from the site of mechanical stimulation (black cross).

Time traces from individual ROIs were analyzed offline with custom-made scripts in C/C++. The 360/380 nm fluorescence ratio was used to assess the Ca^2+^ concentration changes in the cytoplasm. First, all extracted Ca^2+^ traces were smoothed by applying an adjacency averaging procedure, in order to reduce the noise. Then, we defined three characteristic times for the characterization of the [Ca^2+^]_i_ signaling in individual LECs, as indicated in Figure 1a. The response time of the *i*-th cell, *t*_res,*i*_, symbolizes the time at which the cells started to respond to the mechanical stimulation. The time at which the maximal amplitude of [Ca^2+^]_i_ was reached is labeled with *t*_max,*i*_. Lastly, the time *t*_half,*i*_ symbolizes the half decay time, i.e., the time necessary for the 360/380 ratio to decay by 50% from the maximum towards its pre-stimulation value. These three times were then used to define the [Ca^2+^]_i_ signaling characteristics by means of the activation time, $\Delta t_{\mathrm{act},i}=t_{\max,i}-t_{\mathrm{res},i}$, i.e., the time necessary for the signal to increase from the basal value to its maximal value, and by means of the signal duration time, $\Delta t_{\mathrm{dur},i}=t_{\mathrm{half},i}-t_{\mathrm{res},i}$, i.e., the time necessary for the signal to increase from the basal value to its maximal value, and then decrease its by 50% from the maximum towards its pre-stimulation value, as described previously [13]. Moreover, we used the absolute difference in fluorescence ratio between the basal value and maximal value as the estimation for the amplitude of the Ca^2+^ signal. In our analyses, we predominantly investigate the characteristics of Ca^2+^ signaling with regard to the distance from the point of mechanical stimulation. More specifically, we calculated the average response times, average activation times, the average signal durations, and the average relative amplitude for all cells located in ring (annulus) with a given radius. The difference between the larger and the smaller circle defining the annulus was always 25 µm. In this manner, we calculated the signal characteristics for all cells that were present with respect to stimulation point: (1) closer than 25 µm, (2) between 25 µm and 50 µm away, (3) between 50 µm and 75 µm away, etc., as illustrated in Figure 1b.

### 2.5. Statistical Analysis

A standard *t*-test was applied to test the statistical differences between two subgroups referring to different stages of the cataract if data was normally distributed, whilst a Mann–Whitney rank-sum test was used if data were not normally distributed. The statistical significance between three subgroups referring to different types of cataract was evaluated with a one-way ANOVA test. If data failed the normality test, the Kruskal–Wallis ANOVA test on ranks was used instead. For all analyses, *p* < 0.05 was considered as statistically significant.

## 3. Results

We systematically analyzed different aspects of intra- and intercellular Ca^2+^ signaling in the human anterior lens epithelium, built from LECs, whereby we compared different signaling characteristics with regard to the degree of cataract progression, where 1 was the lowest and 5 was the highest level of cataract development, and we considered the type of the cataracts as C, N, and C&N. The former encompasses the comparison between mild (CN1–2) and severe (CN3–5) cataracts, irrespective of the type of cataract. The results were based on 17 LC from mild cataracts (7 C, 7 N, 3 C&N) and 13 LC from severe cataracts (3 C, 7 N, 3 C&N). The experimental protocol consisted of the time before stimulation, the time of mechanical stimulation, and the subsequent recovery time. 

**Figure 2:**
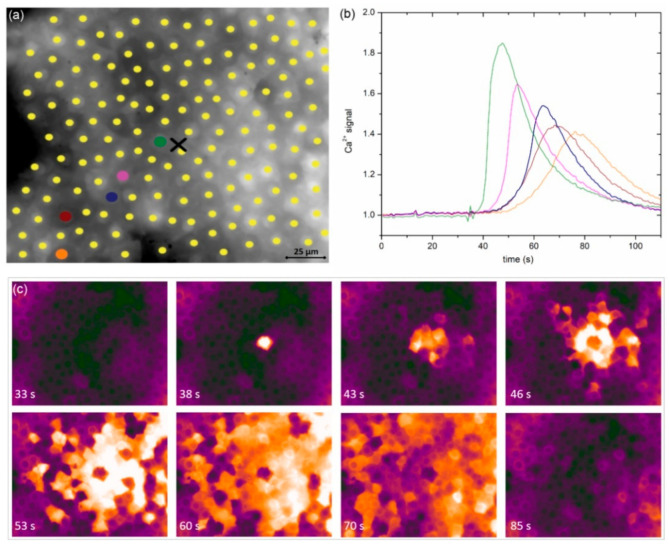
Spatiotemporal calcium response of LECs from a typical mild (C1) cataract to mechanical stimulation. (**a**) Selected LECs, i.e., midpoints of ROIs, superimposed on the anterior LC epithelium image and recorded with 360 nm. The black cross shows the location of mechanical stimulation. (**b**) The time courses of the 360/380 ratio, proportional to [Ca^2+^]_i_, for selected cells. Colors of ROIs in (**a**) correspond to colors of time traces in (**b**). (**c**) A series of 360/380 ratio images at the indicated time points. The second ratio image shows the moment of mechanical stimulation. The values of the ratio are color coded with black/purple, representing low ratio values and corresponding to the low [Ca^2+^]_i_, and orange/white, representing high ratios and corresponding to the high [Ca^2+^]_i_.

In Figure 2a, we show an image of a typical anterior LC’s epithelium from a typical mild cortical cataract (C1) with the indicated centers of LECs (midpoints of regions of interest, i.e., ROIs). In response to local mechanical stimulation (black cross indicates the position), the cells responded with a transient increase in Ca^2+^ (Figure 2b) that propagated radially outwards from the stimulation site in the form of a Ca^2+^ wave (Figure 2c). Notably, not only did more remote cells respond with significant time delays due to signal propagation, but the characteristics of intracellular Ca^2+^ signal, i.e., the amplitude, transient duration, and the slope of Ca^2+^, also increased and appeared to differ with respect to the distance from the stimulation point. We address this issue in more detail in later in this paper.

**Figure 3:**
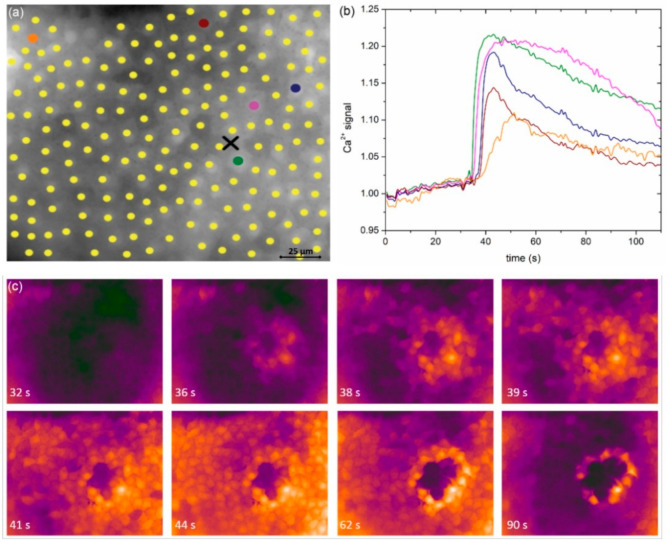
Spatiotemporal calcium response of LECs from a typical severe (C3) cataract to mechanical stimulation. (**a**) Selected LECs, i.e., midpoints of ROIs superimposed on the anterior LC epithelium image recorded with 360 nm. The black cross shows the location of mechanical stimulation. (**b**) The time courses of the 360/380 ratio, proportional to [Ca^2+^]_i_, for selected cells. Colors of ROIs in (**a**) correspond to colors of time traces in (**b**). (**c**) A series of 360/380 ratio images at the indicated time points. The second ratio image shows the moment of mechanical stimulation. The values of the ratio are color coded with black/purple, representing low ratio values and corresponding to the low [Ca^2+^]_i_, and orange/white, representing high ratios and corresponding to the high [Ca^2+^]_i_.

In a LC from a typical severe cortical cataract (C3), very similar results were obtained conceptually and are presented in Figure 3. Again, the signal propagated radially outwards from the stimulation site and the characteristics of Ca^2+^ signals seemed to be location-dependent. A visual assessment of the results in Figure 2 and Figure 3 indicates that the signal propagation is faster in the case of a severe cataract, and, in addition, the duration of Ca^2+^ transients is longer, whereas their relative amplitude is lower. Moreover, a more precise progress of the spatiotemporal Ca^2+^ activity after localized mechanical stimulation is visualized in Videos S1 and S2 for the presented LC from the C1 and the C3 cataracts, respectively.

**Figure 4:**
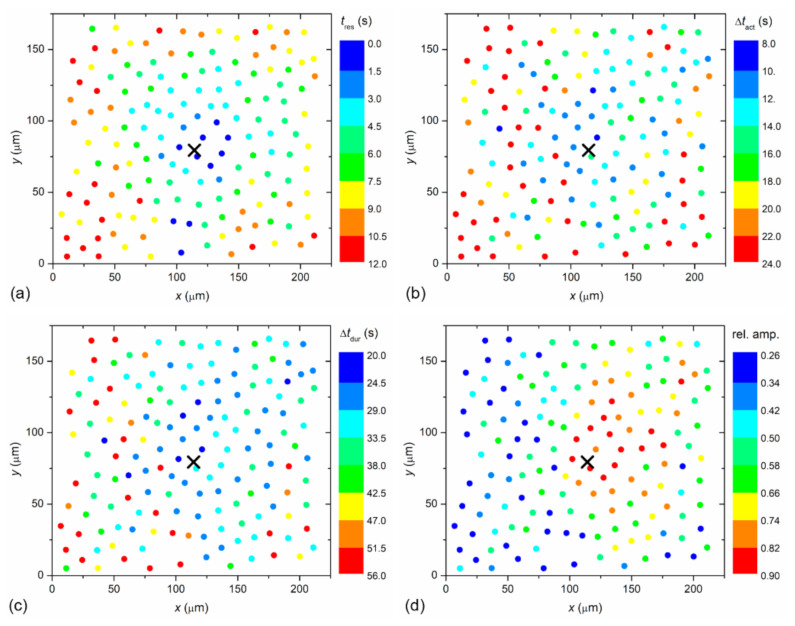
Visualization of the spatiotemporal activity of LECs in a LC from a mild cataract (C1). The location of circles refers to the physical positions of LECs and they are color coded in accordance to their: response times, *t*_res_ (**a**); activation times, Δ*t*_act_ (**b**); signal duration times, Δ*t*_dur_ (**c**); and the relative amplitudes of calcium signals (**d**). Blue color indicates the lowest value, red indicates the highest. The exact values are given in the color bars, separate for each panel.

**Figure 5:**
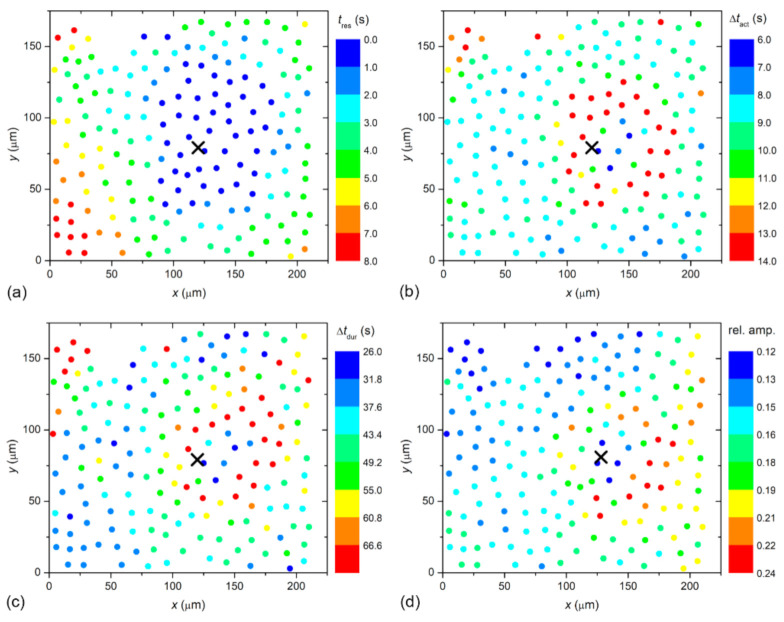
Visualization of the spatiotemporal activity of LECs in a LC from a severe cataract (C3). The location of circles refers to physical positions of LECs and they are color coded in accordance to their: response times, *t*_res_ (**a**); activation times, Δ*t*_act_ (**b**); signal duration times, Δ*t*_dur_ (**c**); and the relative amplitudes of calcium signals (**d**). Blue color indicates the lowest value, red indicates the highest. The exact values are given in the color bars, separate for each panel.

To visualize the spatiotemporal Ca^2+^ responses more precisely, we present the color-coded values of times of interest for the particular LC from a mild (C1; from Figure 2) and a severe (C3; from Figure 3) cataract, respectively, in Figure 4 and Figure 5. More precisely, each colored dot represents an individual cell, i.e., the center of ROI, whereas the color signifies the value of a given signaling attribute, as indicated by the color bars (see Materials and Methods and Figure 1 for details). As expected, in both cases, the response times of cells *t*_res_, i.e., the time at which a particular cell started to respond with a rise in [Ca^2+^]_i_ after stimulation, increased with increasing distance from the stimulation, but the signal spreading was faster in the LC from a severe cataract (see Figure 4a and Figure 5a). On the contrary, the relative amplitude in both LC’s decreased with increasing distance from the stimulation, and the relative amplitudes of Ca^2+^ responses were in general higher in the LC from the mild cataract (see Figure 4b and Figure 5b). Moreover, a distinguishing spatial pattern could be inferred neither in the activation times Δ*t*_act_ nor in signal duration times Δ*t*_dur_ in the LC from a mild cataract. In contrast, in the LC from the severe cataract, there appeared to be a tendency that the cells in proximity of the stimulation point exhibited slower Ca^2+^ responses, even though they were also slowly responding to cells noted in the non-central regions.

**Figure 6:**
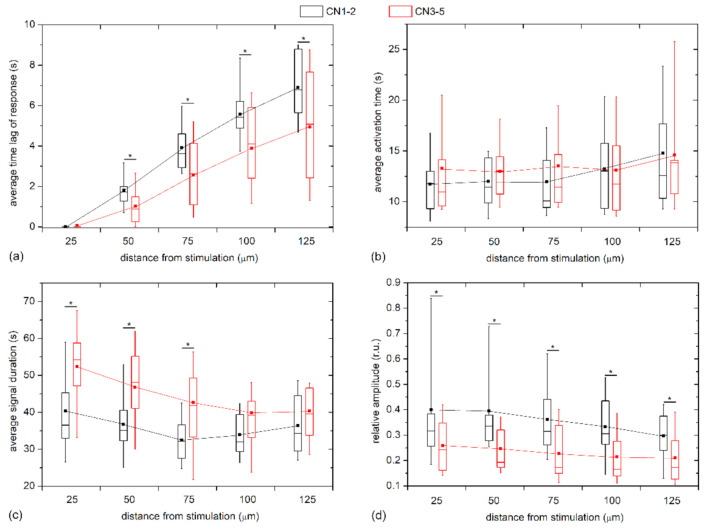
Quantification of calcium signaling in LC LECs with respect to the degree of cataracts. Differences in the response times, *t*_res_ (**a**); activation times, Δ*t*_act_ (**b**); duration times, Δ*t*_dur_ (**c**); and relative amplitudes (**d**) for mild (CN 1–2, black) and severe (CN 3–5, red) cataracts in relation to the distance from the point of stimulation. Boxes determinate the interval within the 25th and 75th percentiles, whiskers denote the 10th and the 90th percentiles, lines within the boxes indicate the median, and the small squares stand for the average value. Lines connecting the averages are plotted to indicate the radial trend of particular measures from the stimulation site.

To quantify the visually inferred findings from the previous figures, and to reckon the general LEC behavior from multiple LCs and examine its dependence on the distance from stimulation, we plotted different Ca^2+^ signaling characteristics in different distance intervals separately for mild cataracts (17 LC; 7 C, 7 N, 3 CN) and severe cataracts (13 LC; 3 C, 7 N, 3 CN) in Figure 6. In particular, the boxplots encompass the average values of different LCs based on all cells in the given distance interval, i.e., the annulus (see Materials and Methods and Figure 1 or details). In general, all properties of Ca^2+^ transients followed a very similar trend in both groups. However, the results in Figure 6a reveal that the time lags after the mechanical stimulation reflected by the average response times, *t*_res_, were more pronounced in the LC from mild cataracts, thereby indicating that the signal propagates more rapidly in LCs from more developed cataracts. To be more precise, the Ca^2+^ waves were on average roughly 30% faster in LCs from severe cataracts. On the other hand, no significant differences were inferred by the activation times, Δ*t*_act_, of LECs at any distance (see Figure 6b). In contrast, the evaluation of the average signal durations, Δ*t*_dur_, indicated that the Ca^2+^ transients lasted longer in LECs originating from more severe cataracts, although the difference faded with increasing distance from the stimulation site. Moreover, in both groups there was a tendency of longer signal durations closer to the stimulation point (Figure 6c). Lastly, we show in Figure 6d the relative amplitudes as a function of distance from the stimulation site. Evidently, the signal amplitudes essentially decreased with increasing distance from local stimulation, and, most importantly, the amplitudes in the groups of LCs from severe cataracts were significantly lower, on average, they were even up to more than 50% lower.

**Figure 7:**
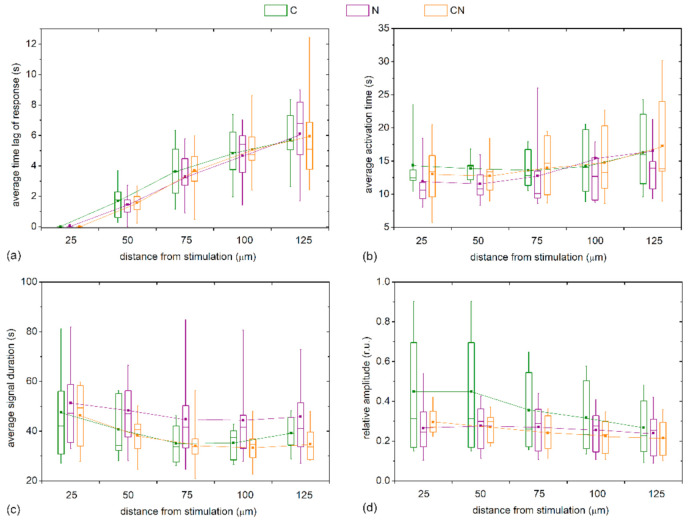
Quantification of calcium signaling in LC LECs with respect to the type of cataracts. Differences in the response times, *t*_res_ (**a**); activation times, Δ*t*_act_ (**b**); duration times, Δ*t*_dur_ (**c**); and relative amplitudes (**d**) for cortical (C, green), nuclear (N, purple) and combined (C&N, orange), cataracts in relation to the distance from the point of stimulation. Box charts are defined the same as in Figure 6.

We also checked whether the Ca^2+^ signaling attributes depend on the type of the cataract. For this purpose, we present in Figure 7 the characteristic signaling times for different distance intervals from the stimulation site separately for different types of cataract (C, N, and C&N). Evidently, the trends were very similar for all four examined signaling characteristics and the intercapsule variability was rather high. Most importantly, significant differences were not detected in any of the characteristics at any distance. Apparently, the Ca^2+^ signaling after mechanical stimulations depends on the stage of the cataract and not the type.

**Figure 8:**
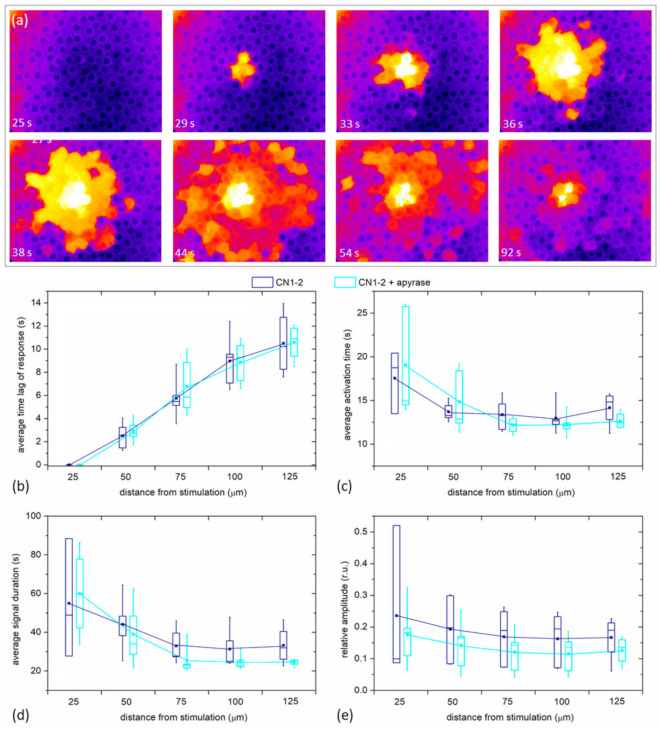
The effect of Apyrase on the spatiotemporal calcium responses of LECs to mechanical stimulation. (**a**) A series of 360/380 ratio images at the indicated time points. The second ratio image shows the moment of mechanical stimulation. The values of ratio are color coded with black/purple representing low ratio values, corresponding to the low [Ca^2+^]_i_, and orange/white representing high ratios, corresponding to the high [Ca^2+^]_i_. The experiment was performed in a N2 cataract after the LC was incubated for 30 min with Apyrase. Panels (**b**–**e**) show different calcium signaling parameters depending on the distance from the stimulation point for five different LCs in the control experiments and the subsequent incubation with Apyrase. For each LC, a different part of the lens epithelial tissue was selected for the second stimulation after incubation. Box charts are defined the same as in Figure 6.

Finally, we examined if the spreading phenomenon depends on paracrine ATP. In Figure 8a we show, similarly as in Figure 1c and Figure 2c, the spreading of Ca^2+^ after the mechanical stimulation, in this case, after the LC was incubated for 30 min with Apyrase, the enzyme that hydrolyzes ATP and effectively removes ATP from the extracellular milieu. In this case, we can observe a very similar spatiotemporal response to the mechanical stimulation as was seen in the control experiments without apyrase. A more quantitative analysis is provided in Figure 8b, where all four signaling parameters (*t*_res_, Δ*t*_act_, Δ*t*_dur_, and the relative amplitude) are shown for five different LCs with mild cataracts. For each LC, two experiments were recorded: first, the control mechanical stimulation response, and then, after the incubation with Apyrase, the second mechanical stimulation response. There was no significant difference in any of the parameters between Apyrase and the control group, indicating that paracrine ATP does not considerably affect the characteristics of Ca^2+^ signals in LCs.

## 4. Discussion

In the present paper we systematically studied the spatiotemporal organization of Ca^2+^ signaling in human postoperative anterior LC’s LECs with the aim to gain further insight into its relation to cataract formation. More specifically, we studied the Ca^2+^ signaling characteristics after local mechanical stimulation with special focus on the role of the degree of cataract progression. Our results revealed that a point of mechanical stimulation induced a Ca^2+^ wave that propagated radially outwards from the stimulation site. By tracking the dynamic changes of the intracellular Ca^2+^ concentration in all cells in the lens epithelial monolayer, we characterized different Ca^2+^ signal parameters as a function of distance from stimulation site. It turned out that Ca^2+^ waves propagate faster in LCs from more developed cataracts than in LCs from less severe ones. Moreover, the relative amplitudes of Ca^2+^ transients were found to be always decreasing with increasing distance from stimulation point, but the amplitudes were found to be significantly lower in LCs associated with higher degrees of cataract pathology. The durations of Ca^2+^ transients were found to be longer in LCs from more developed cataracts, which essentially goes on account of a slower Ca^2+^ decay rate, since a significant difference in the time of Ca^2+^ increase (*t*_act_) between both groups was not detected. However, it should be noted that the relative amplitudes of Ca^2+^ transients in LCs from severe cataracts were considerably lower, and hence, the absolute slope of decay in Ca^2+^ was therefore still steeper in LECs associated with less developed cataracts. With regard to the cataract type, we found no significant differences between the two most frequently present types of cataract, i.e., the cortical, C, and nuclear, N, cataracts, in any examined aspect of Ca^2+^ signaling after mechanical stimulation. Notably, these findings partially parallel our previous investigation about the general Ca^2+^ signaling characteristics after stimulation with ACh, where no differences between different types of cataracts were detected as well [13]. However, in our previous study utilizing a physiological stimulation with ACh, no global Ca^2+^ waves were evoked, which are an important hallmark of epithelial tissues. For that reason, here we used local mechanical stimulation, which facilitated a detailed analysis of the intercellular signalization patterns. By this means, we not only identified that the velocity of Ca^2+^ waves was higher in LCs from severe cataracts, but also that, in contrast to what was observed after the stimulation with ACh, the intracellular Ca^2+^ signaling characteristics were different with respect to the stage of the cataract.

Notably, Gupta et al. [9] also reported that irrespective of the type of cataract, total Ca^2+^ levels are always considerably higher in the LECs from the lenses with the cataract than in those taken from the clear controls. One limitation of our study is that we did not have the clear control, healthy lens epithelia, as our material was obtained after cataract surgery, and thus, we did not have excess to either the clear lens epithelia from cadavers nor to lens from the vitreous surgery. However, having LCs from different degrees of the cataract, based on the modified LOCS III system [22], where 1 is the lowest (almost clear lens) and 5 is the highest level of cataract development, provided some implications about the modulations related to cataractogenesis. We can therefore hypothesize that the Ca^2+^ levels are further increased in LECs from more severe cataracts than from mild cataracts. The velocity of Ca^2+^ waves reflects the functional connectivity and communication between the LECs. For normal functioning of the lens epithelium and the lens, it must be kept tightly regulated. The faster propagation of Ca^2+^ waves in more developed cataracts suggests that the homeostatic mechanisms are more damaged in more developed cataracts, losing the regulation of intercellular Ca^2+^ signal propagation.

At a first glance it seems a bit counterintuitive that the mechanically induced intercellular Ca^2+^ signal propagated faster among LECs from more severe cataracts, whereas, on the other hand, the intracellular Ca^2+^ dynamics was considerably slower. We argue that this observation might be a consequence of higher basal Ca^2+^ levels, which are associated with cataract formation [7,9], and might be even higher in more severe cataract compared to mild cataracts, as discussed above. Accordingly, the threshold for LEC activation due to inputs of neighboring LECs can be reached easier, and hence the propagation is faster. Moreover, this could be related with our observations that the relative signal amplitudes are lower in the case of severe cataracts. It should be noted that our experimental setting did not account for the exact cytoplasmic Ca^2+^ concentration, but the fact that the 360/380 nm ratio increased less in LCs from severe cataracts implies that the basal Ca^2+^ levels were higher. The relative changes in the amplitude reflect the disturbances in the mechanisms that lead to [Ca^2+^]_i_ increase in the cell, be it via Ca^2+^ influx channels or release from intracellular stores, which are very tightly regulated and are an integral part of Ca^2+^ signaling under normal circumstances [7]. With an increased degree of cataract progression, the changes of the amplitudes of Ca^2+^ signals are lower, suggesting that Ca^2+^ homeostasis is more disturbed and its regulation more damaged in more developed cataracts. This seems to be mostly due to the damaged regulation of [Ca^2+^]_i_ decrease in the LECs, as there was no significant difference in the activation time, but there was in the duration time. In more developed cataracts there is most probably more intracellular Ca^2+^ due to its lower extrusion from the cytoplasm, which was also reflected and visible after mechanical stimulation in our study.

Our understanding of the dynamics of spatiotemporal cell signaling were enhanced by analysis in experimental systems that recreated in vivo environments, where cells respond to an extracellular environmental cue as a local mechanical stress. Extracellular mechanical events are translated and processed into intracellular biochemical events. If this mechanochemical translation is impaired, resulting deficiencies in cellular mechanosensation can have an effect on pathologies, as the cataract is.

Mechanical stimulation of individual cells is an established method to study intercellular communication. Ca^2+^ wave propagation triggered by local mechanical stimulation can be used for studying gap junctions and hemichannels [24]. Mechanical stimulation causes a rapid increase in [Ca^2+^]_i_, which, in the form of a wave, extends from the mechanically stimulated cell to adjacent cells. Ca^2+^ signal propagation is studied in many different tissues, reflecting its importance in their functionality. The first major reports of intercellular Ca^2+^ waves following mechanical stimulation of a single cell appeared in 1990, and described Ca^2+^ waves propagating through cultured airway epithelial cells [25,26]. Subsequently, intercellular Ca^2+^ waves have been found to be initiated by a mechanical stimulation in a diversity of cell types, including cultured ovine LECs [19], cultured bovine corneal endothelial cells [27], cultured human retinal pigment epithelium cells ARPE-19 [28], cultured human umbilical vein endothelial cells [29], rat osteosarcoma cell line [30], human umbilical vein endothelial cells [31], avian tendon cells [32], glial cells [33], neurons [34], astrocytes [35], and many others. To the best of our knowledge, most studies utilizing mechanical stimulation, except from some neurological endeavors [33], have been performed with monolayer cell cultures and not postoperative tissue with preserved intercellular connections.

Mechanical stimulation is proposed to result in membrane stress that triggers the production of inositol 1,4,5-trisphosphate (IP_3_) in the stimulated cell [25]. IP_3_ serves as an agonist of Ca^2+^ release from the endoplasmic reticulum and underlies dynamic [Ca^2+^]_i_ changes, including Ca^2+^ oscillations [36]. IP_3_-mediated Ca^2+^ release has also been demonstrated in LECs [37]. A disadvantage of mechanical stimulation is that it may lead to plasma membrane disruption; this would allow for both Ca^2+^ entry into the cell and the liberation of cell constituents, such as ATP or other messengers, from the cell [25].

In response to various types of external changes, including mechanical stress, as well as shear, ionic, and ischaemic stress, hemichannels CxHCs open. Gap junction channels facilitate the transfer of ions and molecules up to 1 kDa among coupled cells [4,38,39]. In addition to gap junction channels, connexins may form hemichannels (CxHCs), termed “connexons”, which provide a pathway for cellular communication on their own, independent of their role as structural precursors of gap junctions, namely, those between the cytosol of an individual cell and its extracellular environment. CxHCs provide a pathway for the release from cells of ATP, which act as paracrine messengers by activating purinergic receptors on neighboring cells. This forms the basis of intercellular Ca^2+^ signal propagation, complementing that which is occurring more directly via gap junctions. It was shown that CxHcs open when cells are subjected to point mechanical stimulation of a single cell [40,41]. A novel class of connexin-like proteins was discovered in 2000, namely the pannexin (Panx) family, which gather in a configuration reminiscent of CxHCs, but do not form gap junctions [42]. Pannexin channels facilitate paracrine communication, mainly by controlling the extracellular exchange of ATP, cyclic AMP, IP_3_, and Ca^2+^. The regulated ATP release through PanxHCs HCs is implicated in a number of normal physiological functions and in response to stressors or pathological states in cells and tissues [43,44,45]. Notably, our results (Figure 8) indicated that the Ca^2+^ spreading phenomenon in the human anterior lens epithelium is not governed by the paracrine ATP effect, thereby identifying the gap junctional communication as the key intercellular signaling mechanism in LECs.

To conclude, we demonstrated that the localized mechanical stimulation of human postoperative anterior LC epithelial cells induced a wave of increased Ca^2+^ that was communicated to surrounding cells. Irrespective of the type or stage of the cataract, the amplitudes of the Ca^2+^ transients were found to decrease with increasing distance from the stimulation, but with a rather slow decay rate. The spread of the locally induced signal was typically well beyond our field of observation (~125 µm), thereby substantiating an important role of intercellular signaling mechanisms between LECs for the normal functioning of the lens. Importantly, significant changes in Ca^2+^ signalization were obtained when comparing LC from different stages of the cataract. Our findings thus indicate that cataract progression entails the impairment of specific Ca^2+^ signaling pathways, which opens some new issues that will need to be addressed during future efforts within lens pathophysiology research. Moreover, we also showed that the paracrine ATP-mediated pathway does not affect the spatiotemporal Ca^2+^ signaling characteristics, indicating the predominant importance of gap junctions in intercellular signaling and communication in human anterior lens epithelium.

## Figures and Tables

**Table 1:** Information on patient and lens capsules data.

Patient No.	Gender	Age	Cataract Type	Cataract Degree	No. of Experiments
1	F	69	nuclear	2	5
2	F	35	nuclear	1	2
3	M	68	nuclear	/	3
4	F	76	nuclear	5	2
5	M	72	nuclear	3	3
6	F	75	nuclear	3	2
7	F	81	nuclear + cortical	2	1
8	F	82	nuclear + cortical	2	2
9	M	57	nuclear + cortical	3	2
10	M	78	nuclear + cortical	4	2
11	F	57	cortical	1	2
12	M	66	cortical	1	5
13	M	75	cortical	3	3
14 *	F	78	nuclear + cortical	2	1
15 *	F	63	nuclear	/	1
16 *	F	82	cortical	2	1
17 *	M	65	cortical	2	1
18 *	F	83	cortical	2	1

* Material from these patients was used for experiments with Apyrase.

## Data Availability

The data presented in this study are available from the corresponding author (S.A.) upon reasonable request.

## Supplementary Materials

The following are available online at https://www.mdpi.com/article/10.3390/life11050369/s1. Video S1: Calcium activity in LECs reflected by the 360/380 ratio triggered by mechanical stimulation in a LC from a mild cataract (C1). The values of ratio are color coded with black/purple, representing low ratio values and corresponding to the law [Ca^2+^]_i_, and orange/white, representing high ratios and corresponding to the high [Ca^2+^]_i_. Video S2: Calcium activity in LECs reflected by the 360/380 ratio triggered by mechanical stimulation in a LC from a severe cataract (C3). The values of ratio are color coded with black/purple, representing low ratio values and corresponding to the law [Ca^2+^]_i_, and orange/white, representing high ratios and corresponding to the high [Ca^2+^]_i_.
